# Amyloid beta dimers/trimers potently induce cofilin-actin rods that are inhibited by maintaining cofilin-phosphorylation

**DOI:** 10.1186/1750-1326-6-10

**Published:** 2011-01-24

**Authors:** Richard C Davis, Ian T Marsden, Michael T Maloney, Laurie S Minamide, Marcia Podlisny, Dennis J Selkoe, James R Bamburg

**Affiliations:** 1 Department of Biochemistry and Molecular Biology, Colorado State University, Fort Collins, CO 80523-1870, USA; 2 Center for Neurologic Diseases, Brigham and Women's Hospital and Harvard Medical School, 77 Avenue Louis Pasteur, Boston, Massachusetts 02115, USA; 3 Department of Neurology and Neurological Sciences, Stanford University School of Medicine, Stanford, CA 94305, USA

## Abstract

**Background:**

Previously we reported 1 μM synthetic human amyloid beta_1-42 _oligomers induced cofilin dephosphorylation (activation) and formation of cofilin-actin rods within rat hippocampal neurons primarily localized to the dentate gyrus.

**Results:**

Here we demonstrate that a gel filtration fraction of 7PA2 cell-secreted SDS-stable human Aβ dimers and trimers (Aβd/t) induces maximal neuronal rod response at ~250 pM. This is 4,000-fold more active than traditionally prepared human Aβ oligomers, which contain SDS-stable trimers and tetramers, but are devoid of dimers. When incubated under tyrosine oxidizing conditions, synthetic human but not rodent Aβ_1-42_, the latter lacking tyrosine, acquires a marked increase (620 fold for EC_50_) in rod-inducing activity. Gel filtration of this preparation yielded two fractions containing SDS-stable dimers, trimers and tetramers. One, eluting at a similar volume to 7PA2 Aβd/t, had maximum activity at ~5 nM, whereas the other, eluting at the void volume (high-n state), lacked rod inducing activity at the same concentration. Fractions from 7PA2 medium containing Aβ monomers are not active, suggesting oxidized SDS-stable Aβ_1-42 _dimers in a low-n state are the most active rod-inducing species. Aβd/t-induced rods are predominantly localized to the dentate gyrus and mossy fiber tract, reach significance over controls within 2 h of treatment, and are reversible, disappearing by 24 h after Aβd/t washout. Overexpression of cofilin phosphatases increase rod formation when expressed alone and exacerbate rod formation when coupled with Aβd/t, whereas overexpression of a cofilin kinase inhibits Aβd/t-induced rod formation.

**Conclusions:**

Together these data support a mechanism by which Aβd/t alters the actin cytoskeleton via effects on cofilin in neurons critical to learning and memory.

## Introduction

Proteolytic cleavage of amyloid precursor protein (AβPP) by β- and γ-secretases gives rise to Aβ peptides ranging in length from 36-43 amino acids [1-6]. Early onset familial AD is linked with high penetrance to mutations that lead to increased production of the most amyloidogenic species, Aβ_1-42 _[4,7-10]. The "amyloid hypothesis" proposes that increasing cerebral accumulation of Aβ over years to decades exacerbates cognitive decline, neurodegeneration, and senile plaque deposition associated with AD. Elevated Aβ can result from mutations or allele expression patterns (or both) that enhances its production/aggregation or decreases its clearance/degradation [2].

The concept that different isoforms and/or conformations of Aβ deliver independent signals to neurons is widely supported [11]. Although the term Aβ is used to describe a spectrum of peptide species, the effects of different Aβ peptide species on neuronal function or morphology are not the same [12,13]. Emphasis has been placed recently on the characterization of small soluble oligomeric forms of Aβ, sometimes referred to as Aβ-derived diffusible ligands (ADDLs) [14]. Unlike synthetic Aβ peptide oligomers or fibrils, which generally are used at μM or greater concentrations, ADDLs are toxic to cultured neurons at nanomolar concentrations [15] and at 500 nM they prevent high frequency stimulation-induced long-term potentiation (LTP) [16]. Furthermore, ADDLs have been linked to hippocampus-dependent temporal memory deficits in mice [17,18].

An even more potent synaptic-inhibitory preparation of human Aβ containing SDS-stable dimers and trimers (Aβd/t) has been obtained by gel filtration of culture medium of a CHO cell line (7PA2) expressing a mutant human AβPP [19]. When used at physiologically relevant (sub-nanomolar) concentrations to treat hippocampal slices, the Aβd/t fraction, as well as a fraction containing SDS-stable Aβ dimer extracted from postmortem human AD brain, strongly inhibited the development of LTP and enhanced long-term depression (LTD), electrophysiological correlates of learning and memory defects in intact animals [20,21]. Although the presence of both Aβ monomer and dimer in Tris-buffered saline (TBS) and TBS/Triton extracts of human brain correlates well with Alzheimer-type dementia [22], it is only the fraction containing SDS-stable dimer that has strong inhibitory activity on the establishment of LTP in acute hippocampal slices [21]. Single intracerebral ventricular (i.c.v.) infusions into adult rat brain of either Aβd/t from 7PA2 cells or Aβ dimer from AD brain caused transient memory and learning deficits [19-21,23,24]. Infusion (i.c.v.) of Aβd/t into adult rat brain several hours after training inhibits synaptic remodeling that accompanies learning and memory consolidation by preventing a transient increase in the number of synapses in the dentate gyrus [24]. Although their mechanism is unknown, the SDS-stable Aβ dimer and Aβd/t cause synaptic dysfunction at sub-nanomolar concentrations, which are 10^3^- 10^4 ^fold lower than commonly used traditionally prepared oligomeric forms of synthetic Aβ (Aβsyn) and 10^2^-10^3 ^fold lower than concentrations of ADDLs. Indeed, direct comparison by i.c.v. injection of cell-derived and Aβsyn oligomers on cognitive impairment in rats confirms the large activity differences between peptide species [25].

In addition to the classical hallmarks of AD pathology that include amyloid plaques and phospho-tau-containing neuropil threads and neurofibrillary tangles, histopathological structures involving actin and the actin-binding protein, cofilin, have been identified in AD brain (reviewed in [26]). Rod-shaped arrays of cofilin-saturated actin bundles (cofilin-actin rods) are induced in cultured hippocampal neurons and organotypic hippocampal slice cultures in response to mitochondrial dysregulation (ATP-depletion) [27-30], oxidative stress [27,31], excitotoxic glutamate [27], extracellular ATP [32], overexpression of cofilin [33], and exposure to Aβ oligomers [29,34], each of which is a potential mediator of synaptic loss observed in both familial and sporadic AD (reviewed in [35]). Rods contain actin and cofilin in a 1:1 complex [36], they form in tandem arrays (striations) within neurites, and they serve as sites for accumulation of phosphorylated tau [37,38], suggesting that they may play a role in formation of striated neuropil threads, the major tau pathology in human AD brain. Cofilin-actin rods can grow to completely occlude the neurites in which they form causing microtubule loss [27] and thus inhibit vesicular transport [34,39]. Because an early indication of AD is blockage in axonal transport that leads to axonal swellings [40,41] and synaptic loss [42], we investigated the ability of physiologically relevant amounts of Aβd/t to induce cofilin-actin rod formation in rat hippocampal neurons and organotypic slices. We examined activity of Aβsyn and showed that its oxidation yielded a product that was ~1000 fold more active in rod induction. The dose, time course and reversibility of Aβd/t rod formation, along with the location of rods in the hippocampus, all suggest that cofilin-actin rods are likely mediators of Aβd/t-induced synaptic dysfunction leading to memory and learning deficits.

## Materials and Methods

### Reagents

All chemicals are reagent grade and were obtained from Sigma-Aldrich Co. and all tissue culture reagents were from Life Technologies (Invitrogen, Carlsbad, CA). Synthetic amyloid-β peptide (Aβ_1-42_) and a scrambled peptide with the Aβ_1-42 _amino acid composition were purchased from AnaSpec, Inc. (San Jose, CA). Aβ monomer and dimer/trimer fractions were prepared from the culture medium of CHO 7PA2 cells [19] as previously described [20,43], and unless noted otherwise were used at 1X concentration (equivalent to their secreted concentration in the medium which was determined below). Similar fractions obtained from culture medium of wild type CHO cells were used as controls.

### Culture treatments of dissociated neurons and slices

Synthetic Aβ oligomers were made by solubilizing the peptide in hexafluoroisopropanol and drying in 10 μg aliquots. Each 10 μg of synthetic Aβ_1-42 _was solubilized in 10 μL of DMSO, diluted with 78.6 μL of sterile Ham's F-12 (to yield a 25 μM stock) and incubated 24 h at 4°C [34,44,45]. Scrambled peptide was prepared identically and both scrambled peptide and synthetic Aβ_1-42 _oligomers were added to final concentration of 1 μM. The 7PA2 cell secreted Aβ fractions (monomer or d/t) or the corresponding fractions from control medium, were prepared as described [20] and, after gel filtration, were freeze-dried to remove the ammonium acetate buffer. These were reconstituted to 5X or 10X in culture medium and diluted with culture medium to achieve the desired final concentrations. Peroxide oxidized Aβ_1-42 _dimer was prepared from synthetic human Aβ_1-42 _(5 μM) by incubation in PBS in the presence of hydrogen peroxide plus or minus Cu^2+ ^as previously described [46-48]. After 3 days in peroxide alone or 5 days in peroxide plus Cu^2+^, the mixture was assayed directly for rod inducing activity in dissociated neuronal cultures (see below) or was fractionated by gel filtration using the same column and elution conditions for preparing the 7PA2 Aβd/t. The fractions were also assayed for rod inducing ability and for Aβ content using the dot blot assay (see below).

### Animals

Timed pregnant Sprague Dawley rats were obtained from Harlan (Indianapolis, IN). E18 fetal rat hippocampal neurons were obtained from timed-pregnant dams and were frozen for future culture work as per published methods [49]. Pups were sacrificed on postnatal days 6-10 for slice preparation. Animal studies were performed according to the National Research Council's guide for care and use of laboratory animals using protocols approved by the Institutional Animal Care and Use Committee.

### Dissociated hippocampal neurons and organotypic slice cultures

Primary hippocampal neurons from E18 rat embryos were prepared and cultured essentially as described [27]. Most assays for rod formation were performed on poly-D-lysine coated 8-well chamber slides or on 15 mm round coverslips in 24-well plates, with about 10-15,000 neurons per well. Neurons were cultured for 4 days in Neurobasal + B27 supplements, treated on day 4 with rod-inducing reagents and fixed and stained for cofilin rods on day 5. Hippocampal slice cultures were prepared from P6-P10 Sprague Dawley rat pups as described [50]. Briefly, hippocampi were quickly dissected into filter sterilized ice-cold (4°C) Gey's Balanced Salt Solution plus 4% glucose, then sliced to a thickness of 400 μm on a McIlwain tissue chopper. For some slices we maintained some entorhinal cortex along with the hippocampus to minimize the degeneration of the perforant pathway [29]. For most slice treatments, 3-6 slices were arranged onto 0.4 μm Transwell^® ^Polyester membranes inserted into 6 well culture plates (Corning Costar^® ^3450, Lowell, MA). Beneath the membrane is added 1.7 mL of filter sterilized slice culture medium (50 mL horse serum, 50 mL Hank's Balanced Salt Solution (HBSS), 100 mL Minimal Essential Medium (MEM), 250 μL 200 mM GlutaMAX, 4 mL 25% glucose, 2 mL 1 M 4-(2-hydroxyethyl)-1-piperazine ethane sulfonic acid (HEPES), pH 7.3, 1 mL 10,000 U/mL Penicillin-Streptomycin). Slice culture medium is aspirated and replaced with 1.5 mL of fresh medium on day 3 and every 2-3 days thereafter or with treatment medium as required. For all experiments slices were cultured for about 10 days in a 95% air/5%CO_2 _incubator at 35.5°C.

### Immunoblotting

Lyophilized gel filtration fractions of 7PA2 and control culture medium, and aliquots of synthetic Aβ oligomer preparations were resuspended in 10 μL of 2X sample buffer [51]. For Western blots, samples were electrophoresed on 10-20% acrylamide gradient Tris-Tricine Ready Gels (Bio-Rad, Hercules, CA). Proteins were transferred onto nitrocellulose (0.1 μm; Whatman, Dassel, Germany), the membrane heated to boiling for 10 min in PBS [52], blocked at room temperature in 1% BSA, 2% goat serum in 20 mM Tris-HCl, pH 7.4, containing 150 mM NaCl (TBS) for 30 min, and incubated overnight at 4°C in primary antibody, Aβ monoclonal 6E10 (Covance, Dedham, MA; 1:1000 in TBS plus 0.05% Tween-20 (TBST)).

Blots were incubated in secondary antibodies (DyLight 680 or DyLight 800 conjugates,1:15,000; Thermo Scientific, Rockford, IL) for 45 min at room temperature. Blots were washed with TBST, imaged with a LI-COR Odyssey Infrared Imaging System, and band intensities quantified using TotalLab software (Nonlinear Dynamics, Newcastle upon Tyne, UK).

Aβ was quantified in 7PA2 cell culture medium, combined gel filtration fractions of the 7PA2 medium, synthetic Aβ oligomers, and oxidized synthetic Aβ preparations and their gel filtration fractions using a dot blot assay on 0.1 μm nitrocellulose with freshly solubilized monomeric synthetic human β-amyloid peptide (Aβ_1-42_) as a standard. Once samples were applied and dried, the membrane was boiled 10 min in PBS to expose epitopes in oligomers and then Aβ was detected with 6E10 antibody and spots quantified as monomer equivalents described above for Western blots. The Aβ monomer was shown to be inactive in rod induction but it was often present to various degrees in gel filtration fractions of Aβd/t. Therefore, the amounts of Aβd/t used in the bioassays were calculated from the total immunoreactive Aβ in the fraction (dot blot assay results) times the percentage of the dimer plus trimer species within each sample quantified from scans of the Western blots described above.

### Adenoviral-mediated gene expression

Adenoviruses for expressing slingshot phosphatase 1L (SSH1L), constitutively active LIM kinase (LIMKT508EE), a dominant negative LIM kinase (D470A) and human cofilin-EGFP have all been described previously [53-55]. These were used at a multiplicity of infection (m.o.i.) of 100-300 for infecting dissociated neurons. For infection of organotypic slices, about 10^7 ^adenoviral particles were added directly to the slice culture medium on day 7 and the cultures were returned to the incubator until treated with Aβd/t or control material on day 8 and fixed for analysis on day 10. Slices cultured on membranes were infected with adenovirus by placing a drop of the adenovirus directly on the slice and adding the excess virus to the culture medium below the slice. One to two hours later the liquid on top of the slice was removed and mixed with the culture medium below the slice. This method gave a higher efficiency of infection than if the virus was only added to the medium below the slice.

### Fixation and immunostaining

Dissociated neurons and slices were fixed for 45 min or 2 h, respectively, at room temperature in 4% paraformaldehyde in either cytoskeletal buffer (CBS; 10 mM MES pH 6.1, 138 mM KCl, 3 mM MgCl_2_, 2 mM EGTA pH 7.0, 4% PEG, 0.32 M sucrose) or PBS adjusted to pH 7.0, with no apparent differences between buffers. Neurons and slices were methanol (-20°C) permeabilized for 3 min (cells) or 10 min (slices) and blocked in 2% goat serum/1% bovine serum albumin in TBS before immunostaining. Primary antibodies include: affinity purified rabbit 1439 IgG to chick ADF (2 ng/μL), which cross-reacts with mammalian ADF and cofilin [56], and protein A purified monoclonal mouse anti-cofilin (MAb22; 10 ng/μL IgG) [57]. Secondary antibodies, all used at 1:450, include Alexa 488 goat anti-rabbit and goat anti-mouse and Alexa 594 goat anti-rabbit and goat anti-mouse (Molecular Probes, Eugene, OR). DAPI (4'-6-Diamidino-2-phenylindole) or Hoechst 33342 were used to stain DNA. Slices on membrane were excised, blocked, immunostained, and mounted on 22 × 22 mm cover glasses with ProLong Gold Antifade (Molecular Probes).

### Microscopy and Image analysis

An Olympus IX81 microscope equipped with an ASI piezo stage (Applied Scientific Instrumentation, Eugene, OR), CSU22 spinning disk confocal head (Yokogawa Instruments, Japan), 440 nm, 473 nm and 561 nm diode lasers, and a 1K × 1K Cascade II EMCCD Camera (Roper Scientific, Tucson, AZ), all integrated and operated by SlideBook software (Intelligent Imaging Innovations, Denver, CO), was used to obtain confocal sections through organotypic slices. The objectives used include 4x Fluorite (0.13 NA), UAPO40X/340W-DIC (1.35 NA), or PlanAPO 60x (1.42 NA). Phase-contrast and non-confocal fluorescence micrographs were also obtained on a Nikon Diaphot microscope.

MetaMorph v7.03 software (MDS Analytical Technologies, Toronto, Canada) was used for all digital processing. Following time-lapse imaging the slice was scanned for rod formation. All experiments were repeated a minimum of three times. To ascertain the regional distribution of rods, the total number of rods per field was counted using a 60x oil objective and data from multiple slices were combined onto a schematic of the hippocampus as described previously [29].

### Statistics

Statistical analyses were done with either MATLAB or SPSS v13. Unless otherwise stated, all significance values are at p = 0.05 and all error bars are standard deviations. Any post hoc tests are reported.

## Results

### Aβd/t is a potent inducer of cofilin-actin rods

The total concentration of Aβ species in 7PA2 conditioned medium was previously reported to be 6.4 ng/mL (1.4 nM) based on ELISA [20]. However, oligomers of human Aβ are inefficiently measured by ELISA [58]. Therefore we measured the total human Aβ concentration in several batches of 7PA2 medium using a dot blot assay and obtained the value of 8.3 ± 0.8 ng/mL (Additional file 1). The amounts of Aβ dimer/trimer (Aβd/t) and Aβ monomer (Aβm) in their respective pooled fractions from gel filtration chromatography (Additional file 1) were also determined directly from dot blot assays to be approximately 1.1 ng/mL and 3.6 ng/mL, respectively, equal to about 250 pM and 800 pM (expressed as monomer equivalents). This value for Aβd/t concentration is close to that estimated from the Aβd/t immunostaining on Western blots after epitope exposure by boiling the membrane [20], and is also quite similar to the concentration of Aβd/t in 7PA2 medium estimated by Freir et al.[24].

**Figure 1:**
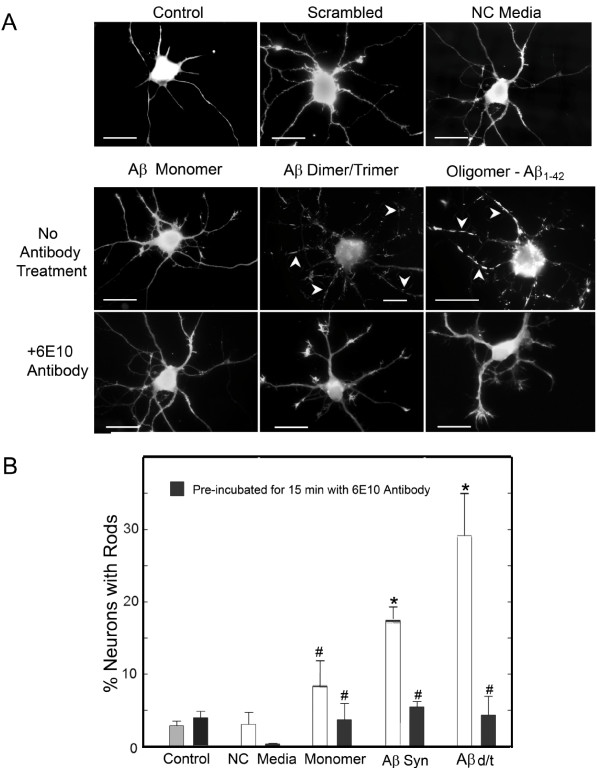
**Aβd/t fraction from 7PA2 cells, but not monomer, induces rods in dissociated hippocampal neurons**. Analysis by fluorescence microscopy of dissociated neurons treated with vehicle (control), scrambled Aβ peptide (1 μM), or non-conditioned (NC media, d/t equivalent fraction), as well as with the monomer and d/t fractions from 7PA2 cell culture medium and 1 μM synthetic Aβ oligomers (Aβsyn). (A) Cofilin immunostained fluorescence images of hippocampal neurons showing representative responses with cofilin-actin rods (arrowheads) formed 24 h after treatment with Aβd/t and Aβsyn oligomers. Pretreatment of the Aβ-fractions with an antibody (6E10) to Aβ eliminates their rod-inducing effects. Bars = 10 μm. (B) Quantification of the rod forming response showing the neutralizing effects of the 6E10 antibody and the non-significant changes in rod formation by Aβ monomer. (* = p = 0.01 compared to control; # not significantly different from control).

Treatment of cultured dissociated hippocampal neurons with the gel fractionated Aβd/t at ~250 pM (1.1 ng/mL; equivalent to the concentration produced in the 7PA2 cell culture medium and referred to as 1X in other references [20,23]) induced the formation of cofilin-actin rods in many neurons, even more than respond to treatment with 1 μM of synthetic human Aβ_1-42 _oligomers (Aβsyn) (Figure 1). Significant rod induction did not occur in neurons treated with either the Aβm or fractionated medium prepared from wild type CHO cells (a control hereafter called non-conditioned (NC) medium). Incubation of Aβd/t or Aβsyn oligomers with 6E10 anti-Aβ monoclonal antibody (as per [20]) for 15 min prior to neuronal treatment reduced rod formation to control levels, implying it is Aβ and not some other component in 7PA2 medium that is responsible for inducing rods. The percentage of neurons with rods was quantified from each of the cultures treated with fractionated NC medium, Aβm, Aβd/t, or Aβsyn oligomers (Figure 1B). Only Aβd/t and Aβsyn oligomers induced a significant (p = 0.01) increase in the percentage of neurons with rods above control (combined data from untreated and synthetic scrambled Aβ peptide-treated samples) and NC media (another control). Pretreatment with 6E10 antibody eliminated this increase. Furthermore, the percentage of Aβd/t-treated neurons forming rods (about 30%) represents a significant (p = 0.05) increase versus treatment with the 1 μM Aβsyn oligomers (18%).

**Figure 2:**
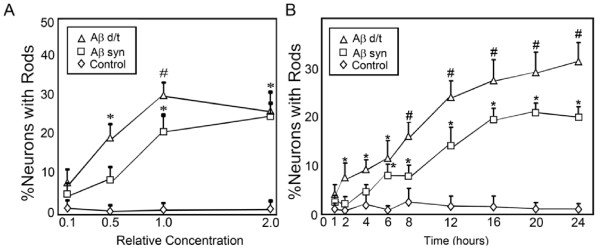
**Percent of neurons in dissociated hippocampal cultures containing rods as a function of Aβ form, concentration and time of treatment**. (A) Dose-response curves for Aβsyn oligomers and Aβd/t versus control. The concentrations are expressed in terms of the 7PA2 CHO cell secreted concentration of Aβd/t (1X = 250 pM), which was used at 0.1, 0.5 and 2X this value. For the Aβsyn oligomers, the 1X value equals 1.0 μM. (B) Following treatment with 1X amounts of Aβd/t or Aβsyn oligomers, neurons were fixed at the times shown and the percent of neurons with rods was quantified. By 2 h the percent of neurons forming rods in response to Aβd/t was significant (*) over controls (p = 0.05). The Aβsyn-treated neurons required 6 h to reach significance over controls. Significance (# = p = 0.05) in the differences between the two Aβ species occurred at 8 h.

We next compared Aβd/t to Aβsyn oligomers for dose-response in rod-induction. The gel filtration fractions containing Aβd/t elute in 50 mM ammonium acetate, pH 8.5 and are freeze dried to remove most of the volatile buffer. However, when reconstituted they cannot be used above a 2.5X concentration because of increased cell death (release of LDH, data not shown). However, neurons could be treated with the Aβd/t from 0.1X to 2X (25 pM to 500 pM) without any significant cell loss over 48 h. Controls were untreated neurons or neurons treated with NC medium. As shown in Figure 2A, the half-maximal response in terms of numbers of neurons forming rods is achieved with ~0.4X Aβd/t (~100 pM) and ~0.7 μM Aβsyn oligomers, about a 7000 fold difference. This value compares favorably with the 4000 fold difference obtained using single point comparison at 1X concentration (250 pM Aβd/t versus 1 μM Aβsyn oligomers), which gives near maximal rod response for each preparation. Furthermore, the maximum percentage of cells with rods is greater with Aβd/t than with Aβsyn oligomers at all concentrations tested (Figure 2A).

### Time course of rod formation

We observed that the percentage of neurons forming rods in response to 1 μM Aβsyn oligomers did not increase significantly over untreated samples until 6 h after addition, as we previously reported [34]. Because the Aβd/t fraction is more active in rod induction than Aβsyn, we directly compared the time course between these two treatments. The Aβd/t fraction at 250 pM induces a measurable increase in rods over untreated controls by 1 h becoming significant (p = 0.05) by 2 h, at which time about 25% of the maximum response is obtained (Figure 2B). By 8 h significantly (p = 0.05) more Aβd/t-treated neurons have rods than neurons treated with 1 μM Aβsyn oligomers and this difference is maintained through the remaining time studied (24 h).

### Oxidation of Aβ_1-42 _generates a rod-inducing SDS-stable dimer

**Figure 3:**
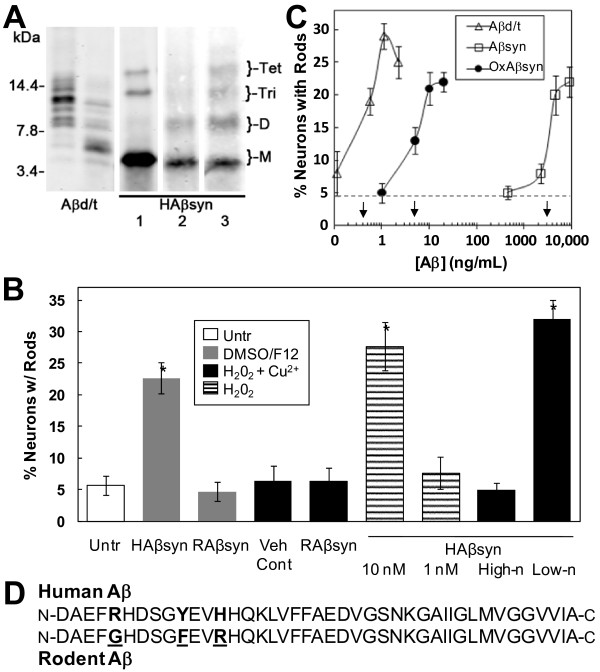
**Oxidized cross-linking of synthetic Aβ**_**1-42 **_**generates a dimer that potently induces rods**. (A) Western blot showing SDS-stable species of Aβ in different preparations. Two gel filtration fractions from medium of 7PA2 cells are combined to give Aβd/t. Last three lanes are synthetic human Aβ_1-42_. Lane 1: traditional oligomers prepared in DMSO/F12 [44,45]; Lane 2: peptide incubated (37°C, 3 d) in PBS containing 250 μM H_2_O_2_; Lane 3: peptide incubated (5 d) in PBS containing 250 μM peroxide plus 25 μM CuCl_2_. Dimer is absent in traditional oligomer preparations but forms in peroxide alone. Dimer, trimer and tetramer are generated with Cu^2+^/peroxide. (B) Comparison of rod-inducing activity between untreated (Untr) dissociated neurons and neurons treated with: Cu^2+^/peroxide (veh cont), 1 μM traditional synthetic human Aβ oligomers (HAβsyn), 1 μM synthetic rodent Aβ (RAβsyn) treated identically to HAβsyn, 1 μM Cu^2+^/peroxide-treated RAβsyn, 10 nM (45 ng/mL) and 1 nM peroxide-oxidized HAβsyn, and 5 nM each of high-n and low-n oligomers-containing fractions of oxidized HAβsyn (see Additional file 3). (C) The rod-inducing activity of different Aβ preparations. The effective concentration for a 50% maximal response (EC_50_; arrows) was calculated from dose-response curves for traditional Aβ oligomers (Aβsyn; ED_50 _= 3100 ng/mL), Cu^2+^/peroxide oxidized synthetic Aβ (OxAβsyn; ED_50 _= 5 ng/mL) and Aβd/t (ED_50 _= 0.4 ng/mL). Dashed line is control (untreated). Bars are standard deviations. (D) Human and rodent Aβ_1-42 _sequences differ in three residues (bold), but only tyrosine at position 10 is likely to generate the peroxide-induced SDS-stable species.

Oligomerization of human Aβsyn is usually performed on peptide solubilized in DMSO [44,45]. These oligomer preparations are noticeably deficient in SDS-stable dimer [59], although SDS-stable trimers and tetramers are present (Figure 3A). Therefore we reasoned that it is likely the increased dimer content that makes the Aβd/t so much more active in rod-induction than Aβsyn. Oxidation methods to prepare stable tyrosine cross-linked human Aβ dimer have been reported [46]. Human Aβ has a single tyrosine at residue 10, which is substituted by phenylalanine in rodent Aβ (Figure 3D). Rodent Aβ (RAβ) does not form dimers or other SDS-stable oligomers on its own [46] and does not induce rods even when used at 1 μM concentration (Figure 3B). Thus, we addressed the possibility that oxidized human Aβ dimers are contributing to the enhanced rod forming activity of the Aβd/t fraction. Synthetic human Aβ_1-42 _was incubated with 250 μM hydrogen peroxide plus or minus 25 μM CuCl_2 _for 3-5 days (Additional file 2). The presence of the CuCl_2 _is required to generate the di-tyrosine dimer [46] but not other oxidation products that include dimers (Figure 3A). We compared the rod-inducing ability of the Cu^2+^/peroxide oxidized synthetic human Aβ (OxAβsyn) to that of the Aβd/t and the traditionally prepared synthetic human Aβ oligomers (Aβsyn) by performing dose-response curves in cultures of rat hippocampal neurons treated on day 4 and fixed on day 5 (Figure 3C). The presence of Cu^2+^/peroxide (Figure 3B) or peroxide alone (data not shown) had no effect on rod formation. It is quite apparent from the curves in Figure 3C that Aβ oxidation dramatically enhances its rod-inducing activity. The effective concentration for half-maximal response (EC_50_) is >7000 fold different between Aβsyn (3100 ng/mL = 690 nM) and Aβd/t (0.4 ng/mL = 90 pM) and >600 fold different between Aβsyn and OxAβsyn (5 ng/mL = 1.1 nM). These concentrations are based on the total Aβ content in the samples tested. If we subtract the amount of monomer remaining in each of the fractions based upon its percentage content from quantified Western blots, there is less than a five-fold difference in the activity of the OxAβsyn and Aβd/t samples. In addition, synthetic rodent Aβ_1-42 _(RAβsyn) is inactive in rod induction at 1 μM when prepared using either standard oligomerizing conditions (DMSO/F12 medium) or Cu^2+^/peroxide treatment (Figure 3B), strongly suggesting that oxidation of tyrosine is what drives much, if not all, of the formation of SDS-stable oligomers. Gel filtration of the Cu^2+^/peroxide-oxidized human Aβsyn produced two peaks containing Aβ (Additional file 3). Western blots showed that both peaks contained similar mixtures of SDS-stable species (monomer, dimer, trimer and tetramer) with about 25% eluting near the void volume (high-n species) and the remaining 75% eluting near the position of 7PA2 cell-secreted Aβd/t (low-n species), which elutes at the position of dimer, based upon globular protein calibration (Additional file 3). The high-n fractions were inactive for rod-inducing activity at 23 ng/mL (5 nM), whereas the low-n fractions at the same concentration gave as strong a response as has been obtained with any Aβ species (Figure 3B). Taken together, these results suggest that a variety of oxidized human Aβ dimers can form, that their formation appears to be dependent upon the tyrosine at position 10, and that their rod-inducing activity may be dramatically impacted by their final aggregation state. The presence of the SDS-stable Aβ dimer in a low-n state appears to be largely responsible for the rod-inducing potency of the Aβ.

### Stability of the added Aβd/t fraction

To determine if the Aβd/t fraction added to the cultured organotypic slices underwent a change in concentration or altered its SDS-stable d/t form during the incubation, we removed culture medium at the end of experiments (48 h). Human Aβ was then immunoprecipitated and reanalyzed by SDS-PAGE (Additional file 4). As observed in previous studies [23], there was little change in the concentration of Aβd/t from what was initially added to the slices.

### Regional analysis of Aβd/t-induced rods in hippocampal slices

**Figure 4:**
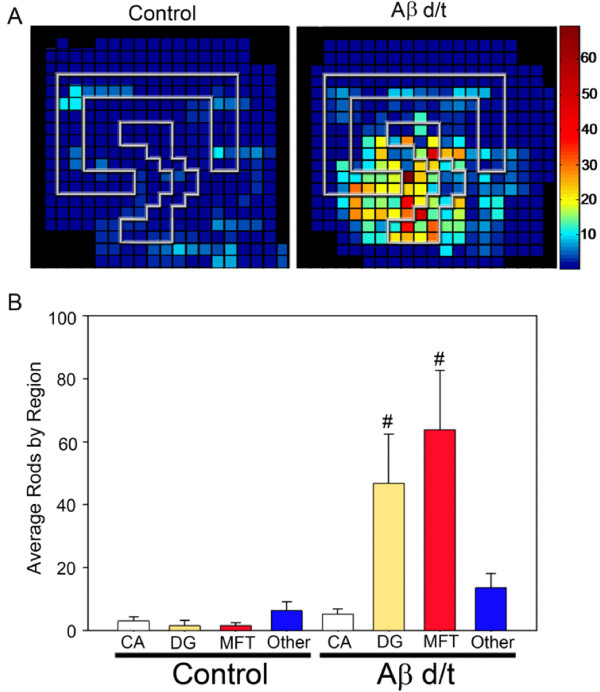
**Numbers of rods induced by Aβd/t are highest in the dentate gyrus and mossy fiber tract**. (A) Organotypic hippocampal slices were cultured for at least 8-10 days and were left untreated or treated with 1X Aβd/t, the same amount of the equivalent NC medium fraction, or scrambled Aβ peptide. After 48 h, slices were fixed and immunostained for cofilin and DNA (DAPI), and rods were quantified by counting with a 60x objective. Rod mapping from multiple slices onto a matrix grid of the hippocampus was performed as previously described using fiduciary markers from the stained nuclei layers to align hippocampal regions [29]. There were no differences detected in rod numbers or distribution between the untreated slices and those treated with NC medium or scrambled peptide and these were all combined to give the control panel. Rods induced by the Aβd/t were heavily concentrated over the dentate gyrus and mossy fiber tract (n = 18 for control slices and n = 12 for Aβd/t treated). (B) Rod quantification averaged per field over different regions of the slices. Each field acquired with the 60x objective has about 6-7 matrix grid squares. The only regions of significance (# = p = 0.05) for the rod numbers are in the dentate gyrus and mossy fiber tract.

The numbers of rods in fields taken from multiple organotypic hippocampal slices that were untreated, treated with NC medium, or treated 48 h with 250 pM Aβd/t were mapped onto a matrix grid of the hippocampus using fiduciary points to stretch and fit multiple slice data onto a single summary map as previous described [29]. Similar to the localization of rods in response to Aβsyn oligomers [29], a treatment also repeated here (data not shown), the Aβd/t-induced rods were mainly localized to the polymorphic hilar region of the dentate gyrus and along the mossy fiber tract into the CA3 region (Figure 4). Furthermore, the numbers of rods per grid square are on average 2-3 fold higher than for the comparable region of the slices treated with Aβsyn oligomer (not shown, but the maximum rods per square on the hot scale in Figure 4A is 60 compared with the maximum of 15 rods per square for synthetic Aβ-treated slices previously published [29]). Rod numbers in slices treated with the gel filtration fraction of NC medium were not significantly different from untreated controls and thus data from these slices were combined to make the control panel (Figure 4A). Similar to what was observed in dissociated neurons (Figure 1B) pretreatment of Aβd/t with 6E10 antibody reduced the rod numbers in slices to control levels (data not shown).

### Dose response curves for rod induction in organotypic slices

**Figure 5:**
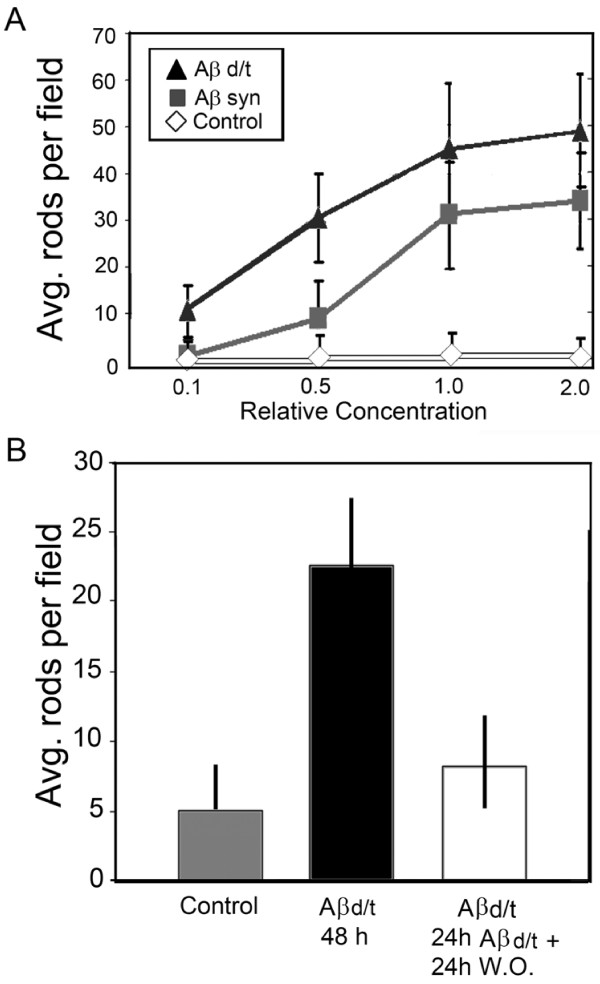
**Dose-response curve for rod formation in organotypic hippocampal slices and reversibility of Aβd/t-induced rods**. (A) The same concentrations of Aβd/t and synthetic oligomer used in Figure 2A were applied to organotypic hippocampal slices. After 48 h, slices were fixed and rods immunostained and quantified per field averaged across the entire slice. The curves obtained are very similar to those in dissociated neurons but the measured parameters are different (average rods per field measured here vs. percent neurons with rods in Figure 2A). (B) Rods formed in response to Aβd/t in hippocampal slices reached their maximum value by 24 h (see Figure 2B). To determine rod reversibility, some of the slices were washed free of the Aβd/t and allowed to incubate another 24 h, whereas others had the Aβd/t present continuously. Controls were treated with the NC medium for 48 h or were left untreated (no difference). As previously shown for the rods induced by synthetic Aβ oligomers [29], the Aβd/t-induced rods are also reversible.

Dose-response curves for rod formation mediated by Aβd/t versus human Aβsyn oligomers in organotypic slice cultures were produced by quantifying rods per field of view (Figure 5A). This measurement is quite distinct from the data in Figure 2A, because here we are counting total rods and not the percentage of neurons with at least one rod. It should be noted that one field of view, using a 60x oil objective, encompasses the equivalent of about 6-7 matrix grid squares and that rod counts were averaged over the entire slice. Slices treated with the Aβd/t fraction at 0.1X (estimated to be 25 pM) still have a significant (p = 0.05) increase in rods per field over controls. Thus, 25 pM Aβd/t induces nearly equivalent numbers of rods per field as 500 nM Aβsyn oligomer. The curves are very similar to the dose-response measured in dissociated neuronal cultures and quantified as percent of neurons with rods (Figure 2A).

### Rods induced by Aβd/t are reversible

To test if rods induced in organotypic slices by Aβd/t are reversible, the Aβd/t was washed out 24 h after treatment and slices were allowed to recover for 24 h in control medium. As previously demonstrated for rods induced by human Aβsyn oligomers [29], the majority of rods induced by 250 pM Aβd/t disappear 24 h after washout (Figure 5B).

**Figure 6:**
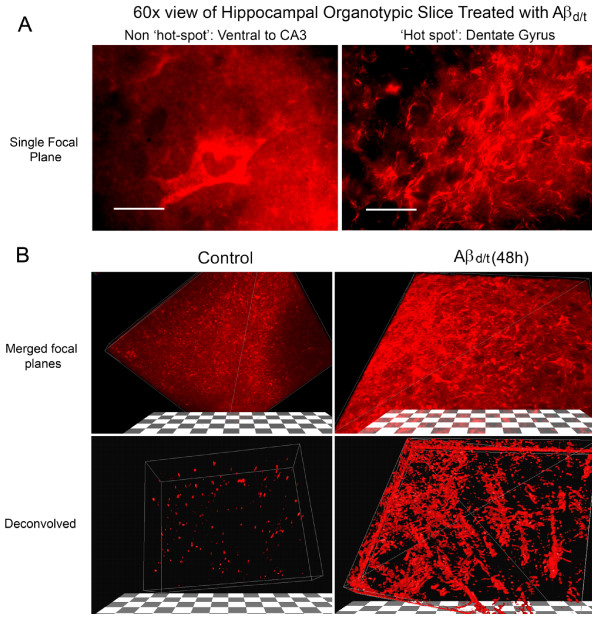
**Three-dimensional reconstruction of cofilin-stained rods in deconvolved confocal image stacks from organotypic slices**. Treatment of organotypic slices with Aβd/t results in a profound increase in cofilin-immunostained rods in the dentate gyrus/mossy fiber tract (DG/MFT) and a global change in cofilin distribution in cells in this region. (A) Single focal plane of non-rod-forming region near the CA3 compared to a rod hot spot in the dentate gyrus. Rods are evident in this single plane. (B) Three dimensional stack of planes from a cofilin stained control and Aβd/t-treated slice. Deconvolution of the confocal image stacks and thresholding the image by removing the lowest 20% of signal (lower panels) provides striking evidence of rod formation in this region. Hundreds of rods can be observed, which contain virtually all of the remaining immunostained cofilin.

Rods are not apparent in low magnification images of slices, but become apparent when viewed with a 60x objective, even in single confocal sections (Figure 6A). However, the rod distribution and abundance within the dentate gyrus are more impressive when the confocal image stack is deconvolved and the lowest 20% intensity of immunstaining is removed by resetting the low threshold on an image histogram (Figure 6B). Only a few densely stained cofilin aggregates are observed in the 3D reconstruction of the image stack from control slices whereas rods are abundant throughout the Aβd/t-treated slice.

### Modulating cofilin phosphorylation alters rod formation and response to Aβd/t

**Figure 7:**
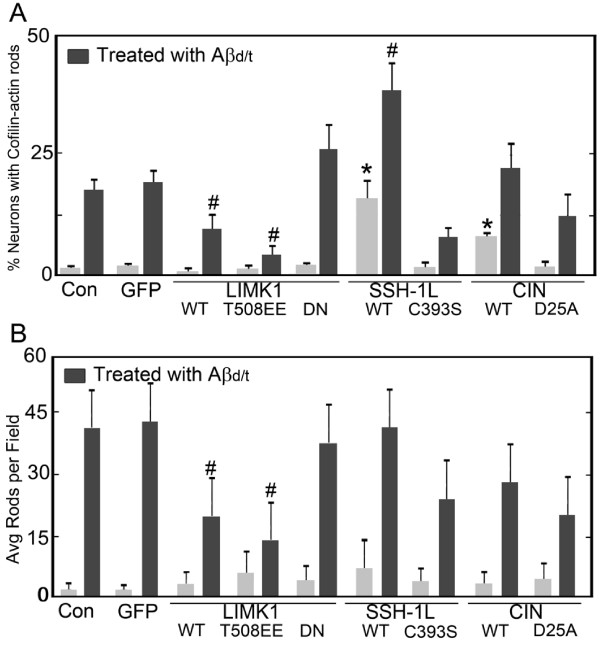
**Upstream regulators of cofilin phosphorylation impact the ability of Aβd/t to induce rods**. Rod formation was quantified in (A) dissociated hippocampal neuronal cultures or (B) organotypic hippocampal slices that were uninfected (Con), infected with control adenovirus expressing GFP (GFP) or with adenoviruses expressing various upstream regulators of cofilin phosphorylation. All viruses co-expressed a fluorescent protein marker and only neurons expressing the marker were scored in the dissociated cultures. In slices, infection rates were approximately 70% (see Additional file 5) and rods per field were quantified (it was not possible in slices to count rods only within infected neurons). Neurons or slices were infected 24 h prior to treatment with Aβd/t (1X) and were fixed and analyzed for rod formation 48 h after Aβd/t addition. Treatments that enhance cofilin dephosphorylation (the active phosphatases SSH-1L WT and CIN WT) in the dissociated cultures enhance rod formation with or without Aβd/t treatment (* = significant difference from untreated or GFP controls at p = 0.05; # significantly different from Aβd/t treated controls, p = 0.05). Treatments that inhibit cofilin dephosphorylation (LIMK1 WT and the active LIMK1T508EE), inhibit rod formation in response to Aβd/t in both dissociated neuronal cultures and in slices (n = 3).

Since rod formation correlates well with cofilin dephosphorylation, we examined the effects of enhancing or inhibiting cofilin dephosphorylation on rod formation in untreated dissociated neuronal cultures or those treated with Aβd/t. Adenoviral-mediated expression of the cofilin phosphatases, either slingshot (SSH1L) or chronophin (CIN), but not their inactive forms, increases rod formation in the absence of any other rod-inducing treatment whereas the inactive form of slingshot (C393S), and less so CIN (D25A), appears to act in a dominant negative manner by reducing rod formation in cells treated with 250 pM Aβd/t (Figure 7A). Fluorescent protein co-expression allowed us to identify the infected cells and score these independently from the uninfected cells in the same culture. Greater than 15% of neurons overexpressing SSH1L formed rods, the most dramatic effect observed, whereas about 8% of neurons expressing CIN formed rods. Treating neurons expressing these cofilin phosphatases with Aβd/t significantly (p = 0.05) enhanced rod formation above the levels induced by the Aβd/t-treatment alone. A maximum of 38% of neurons with rods was measured for the SSH-1L expressing neurons treated with Aβd/t whereas fewer than 10% of neurons expressing the dominant negative form of SSH1L formed rods when treated with Aβd/t.

Expression of a dominant negative LIM kinase, the major cofilin kinase, did not alter rod formation when expressed on its own, however its presence enhanced the percent of neurons forming rods in response to Aβd/t, although the difference (from 19-20% to 26%) is not significant at p = 0.05. Expression of wild type or constitutively active LIMK produced the opposite effect as each significantly (p = 0.05) reduced rod formation in response to Aβd/t by about 50% and over 75% respectively (Figure 7A).

A parallel series of experiments were performed using organotypic slices (Figure 7B). To first estimate the efficiency of transgene expression in the cells of organotypic slices by adenoviral infection, we examined slices at high magnification after adenoviral-mediated expression of a fluorescent protein and staining of nuclei with DAPI. We quantified the number of nuclei with surrounding fluorescent protein expression (Additional file 5) and determined a >70% infection efficiency when we used 10^7 ^infectious particles/slice (based upon a titer of infectious particles per mL obtained by expression of the viral E2a antigen in infected cells [60]). In organotypic slices, expression of SSH1L or CIN alone did not significantly increase rod formation (quantified as rods per field using the 60x objective) over uninfected controls. In addition, in slices infected and expressing SSH1L or CIN, we did not observe an increase in rod formation in response to Aβd/t over Aβd/t-treated uninfected controls. However, in slices infected with virus for expressing LIMK wt and LIMK508EE, the average rod number per field in response to Aβd/t was reduced versus Aβd/t-treated controls (Figure 7B), similar to what we observed in dissociated cells (Figure 7A). Taken together, these results suggest that cofilin dephosphorylation is necessary and may be sufficient to induce rod formation in many neurons but that the total rod response is enhanced by Aβd/t-induced stress.

## Discussion

Here we demonstrate that the dimer/trimer fraction of a naturally secreted form of human Aβ is dramatically more potent in inducing cofilin-actin rods than traditionally prepared human Aβsyn oligomers. We estimate Aβd/t activity is about 4000 fold greater than traditionally prepared human Aβsyn oligomers, but this is based upon an indirect quantification of dimer/trimer concentration using the dot blot to determine total Aβ and gel quantification to estimate the dimer/trimer as a percentage of the total. The maximal activity for Aβd/t (1.1 ng/mL or ~250 pM based on monomer equivalents) is within its estimated physiological concentrations based on the amount extracted from human AD brain [21,22] and the amount detected in cerebrospinal fluid from normal and AD patients [61]. Because the rod-inducing activity of the Aβd/t fraction is neutralized with an Aβ antibody, it is Aβ that appears to be the active rod-inducing component. Furthermore, the lack of significant rod-inducing activity of the Aβ monomer fraction from the 7PA2 medium, even at concentrations three-fold higher than Aβd/t, suggests the SDS-stable dimer is the minimal active unit in promoting cofilin-actin rod formation.

It is not known what is responsible for the size distribution of species within the monomer, and SDS-stable low-n oligomer pools. However, naturally occurring human Aβ species modified at their N-terminus to generate both 3-42 and 4-42 isoforms have been reported [62]. The 3-42 isoform with an N-terminal glutamate is capable of forming the pyroglutamate form, which along with 1-40, 1-42 and 4-42 isoforms, are among the most abundant in control, familial AD and sporadic AD brain [62]. Thus mixed oligomers of these different species probably account for the size distribution observed on Western blots.

The nature of the cross-links to generate the SDS-stable species has also been questioned. It was previously shown that tyrosine10 in the human Aβ sequence can undergo oxidation in the presence of Cu^2+ ^and peroxide to form a specific di-tyrosine cross-link, which did not form in the absence of Cu^2+ ^(or Fe^3+^) [46]. However, other peroxide-induced dimers were produced but not characterized, and these presumably could also result in SDS-stable trimer and tetramer species. Rodent Aβ contains no tyrosine (F10 in place of Y10; Figure 3D), is deficient in formation of SDS-stable oligomers [46,63], and does not induce cofilin-actin rods when used at 1 μM, even after Cu^2+^/peroxide treatment (Figure 3B), suggesting that tyrosine oxidized Aβ and the formation of stable dimers may account for enhanced activity of human Aβ. We investigated the biological activity of oxidized human Aβ prepared with peroxide in the presence and absence of copper [46,47]. Both oxidized human Aβ preparations had greatly enhanced rod-inducing biological activity, with the one made in the presence of copper being active at a concentration within 10 fold of that of Aβd/t from 7PA2 cells. However, the enhanced activity of the human Aβ oxidized in the absence of Cu^2+ ^suggests that species of Aβ dimer (or other SDS-stable low-n oligomers) other than those with a di-tyrosine cross-link also contribute to rod-inducing activity.

We assume that the lack of dimers in traditionally prepared oligomers of human Aβ arises from their rapid assembly at the high concentration of human Aβsyn used [64], combined with the antioxidant effects of DMSO, which is used to solubilize the peptide [44,45]. Our data strongly supports the presence of oxidized human Aβ dimers as contributing to the potent rod-inducing activity of the 7PA2 Aβd/t fractions.

We found that the amount of the Aβd/t did not change in the medium over 3 d. This is due in part to the fact that rodent Aβ does not form SDS-stable oligomers and hence the continued secretion of rodent Aβ by the cultured neurons, which is stimulated by the presence of the human Aβd/t [65], does not contribute to the Aβd/t pool. It also suggests that neuronal uptake of the Aβd/t, which has been reported [66], occurs at a slow enough rate to not significantly deplete the extracellular pool during the 3 day exposure.

Enhancing cofilin's F-actin binding activity by overexpressing either of the cofilin phosphatases, slingshot or chronophin, enhanced rod formation in dissociated neurons in the absence of Aβ-treatment and expression of the cofilin kinase LIMK1 inhibited formation of rods even after Aβ-treatment. This result was not unexpected since overexpression of cofilin, especially the non-phosphorylatable cofilin S3A mutant, enhances rod formation [33]. Expression of LIMK1 in organotypic slices reduced Aβ-mediated rod formation similar to its effect in dissociated neurons. The expression of cofilin phosphatases in organotypic slice cultures produced no significant increase in rod formation either alone or in response to Aβd/t. Although we find about a 70% adenoviral infectivity rate of cells in the slices, it is quite likely that the non-neuronal cells infect better than the neurons; thus for some adenoviruses the neuronal infectivity may be low enough that we would have difficulty in obtaining enough infected neurons to observe significant changes in rod formation.

Single infusions into the adult rat brain of either Aβ dimer extracted from human AD brain or Aβd/t at the identical concentration (250 pM) used in our studies caused transient memory and learning deficits when measured starting 2 h after infusion, and completed within a 2 h maximum time frame [20,21]. Memory and learning deficits disappeared 24 h after the single infusions. It is worthy to note in this regard that the Aβd/t fraction induces rods in a statistically significant number of neurons by 2 h after treatment (about 25% of the maximal response) and that the Aβd/t rods are reversible, disappearing by 24 h after washout. Thus, formation and disappearance of rods in cultured neurons and organotypic slices correlate well with the changes observed in memory and learning in whole animals exposed to a single infusion of Aβd/t.

Nevertheless, the time of rod formation in response to Aβd/t treatment does not correlate well with acute Aβd/t effects on slice electrophysiology. Decreased long-term potentiation (LTP) and enhanced long-term depression (LTD) occur within 20 min of treating hippocampal slices in culture either with fractions containing Aβd/t [23] or with Aβ dimer extracted from human AD brain [21]. This response is more rapid than the 2 h it takes to obtain a significant increase in rods in organotypic slices exposed to the Aβd/t fraction. Rod formation has been observed to occur within 10 min in organotypic slice cultures responding to anoxia or ATP-depletion [27,29]. However, the rate of Aβd/t induced rod formation in organotypic slices maintained in neurobasal/B27 medium may be significantly slower than in acute slices prepared for electrophysiology and maintained in artificial cerebrospinal fluid (aCSF) owing to 0.6 μM insulin in the neurobasal/B27 medium not present in aCSF. Insulin helps neurons resist the pathogenic changes in cytoskeletal organization induced by Aβ [67]. Alternatively or in addition, we suspect that cofilin dephosphorylation and altered actin dynamics in response to Aβd/t is rapid and precedes rod formation in some compartments, such as dendritic spines. Synaptic activity depends directly on cofilin function in regulating actin dynamics and may reflect early changes in plasticity [43,68-70]. Thus, the LTP/LTD response to Aβd/t could be independent of rod formation yet the result of a localized change in cofilin activity. Significantly, wash out of Aβ after the electrophysiological changes have occurred did not result in any reversal in the altered LTP/LTD over 2 h. This persistent effect could arise within the organotypic slice from relatively tight binding of the Aβd/t to specific sites for which some evidence does exist [23,67]. Alternatively, rod formation may have occurred by the time washout was initiated and rods could be responsible for the lack of rapid recovery. Rods sequester most of the cofilin [27,33] needed to re-establish the balance in spine actin dynamics [9] and their formation would resist rapid recoveries of the spine cofilin pool.

The most sensitive pool of neurons forming rods in response to both synthetic Aβ oligomers and Aβd/t are those within the polymorphic/hilar region of the dentate gyrus along the mossy fiber tract into the CA3. The dentate gyrus is considered to play a central role in associative memory [71]. Its major input comes via the perforant pathway with axons representing approximately one million excitatory entorhinal neurons from layer II. These axons end preferentially within the outer two thirds of the superficial molecular layer, mainly on the apical dendrites of the granule cells, but also on dendrites of interneurons [35]. Cholinergic neurons from the basal forebrain provide another important afferent input, and also synapse with neurons of the dentate gyrus inner molecular layer. The CA3 pyramidal cells receive the granule cell output via the mossy fibers (granule cell axons) and aid in pattern completion [72]. Because of its central role in associative memory, the dentate gyrus has been extensively studied in AD brain [35]. There is an early loss of synapses (48% decrease in synapse to neuron ratio) before significant loss of neurons [42,73,74]. Based upon the degree of immunofluorescence labeling for the synaptic marker synaptophysin, there is a direct correlation of synaptic loss during AD progression. Early, mild and severe AD cases are accompanied by a decline in synaptophysin staining of about 25, 45 and 65%, respectively, in the outer and middle third of the molecular layer, with little to no loss in the inner third [75]. Injection of single doses of Aβd/t-containing medium into brains of adult rats also leads to defects in associative memory and to memory consolidation with a striking inhibition of the synaptic increases that occur during memory consolidation in the dentate gyrus [24].

## Conclusions

We have demonstrated that the most synaptotoxic species of Aβ yet identified, that fraction of cell secreted Aβ containing SDS-stable dimers and trimers at sub-nanomolar concentration, is able to induce cofilin-actin rods in neurites of hippocampal neurons. Synthetic human but not rodent Aβ can be converted into this more highly active rod-inducing form by oxidative conditions, suggesting that it is tyrosine oxidation products that are the most active species. Cofilin-actin rod formation in response to Aβd/t is most prominent within the dentate gyrus and mossy fiber tract of the hippocampus. Because rods block transport and cause distal atrophy of the neurites in which they form without death of the neuron [27,39], they represent a likely mechanism to explain the synaptic loss associated with early stages of AD and thus represent a novel target for therapeutic intervention.

## Competing interests

The authors have no competing interests.

## Authors' contributions

This project was conceived by MTM and JRB with input on experimental design and reagents from DJS and MP. RCD took over the project shortly after its initiation and, with technical assistance from LSM, completed most of the neuronal culture and brain slice studies using Aβd/t provided by DJS and characterized by MP. ITM took over Aβd/t purification performed the studies on Aβ characterization, including the oxidation and activity assays of synthetic Aβ. The manuscript was written primarily by JRB with significant input from MTM and other co-authors. All authors have read and approved the final manuscript.

## Supplementary Material

Additional file 1 **Preparation and quantification of Aβd/t**. (A) Aβ Western blot (6E10 antibody) of gel filtration fractions from a single Superdex75 (10/30 HR) column run at a flow rate of 0.5 mL/min and loaded with 1 mL of a 10X concentrate of 7PA2 cell conditioned (16 h) DMEM medium [20,43]. (B, C) Dot blot standard curve for quantification of Aβ monomer equivalents in the 7PA2 culture medium and final Aβd/t fraction. Click here for file

Additional file 2 **Western blot showing time course of oxidative changes in synthetic human Aβ**_**1-42 **_**incubated under different conditions to generate SDS-stable dimers and higher oligomers**. Synthetic human Aβ was dissolved to 5 μM directly into PBS alone or PBS containing 250 μM hydrogen peroxide, 25 μM CuCl_2_, or peroxide plus CuCl_2_, incubated at 37°C, and aliquots were removed at 1 day intervals for the times shown to examine the species present by Western blotting. After transfer, the blotting membrane was heated to boiling to expose the epitopes for detection with the 6E10 antibody. Click here for file

Additional file 3 **Western blot showing fractions of gel filtration column of Cu**^**2+**^**-peroxide-treated synthetic human Aβ**_**1-42**_. Aβ Western blot (6E10 antibody) of gel filtration fractions from a single Superdex75 (10/30 HR) column run at a flow rate of 0.5 mL/min and loaded with 1 mL of the Cu^2+^-peroxide-treated synthetic human Aβ_1-42 _after 5 days of incubation. Fraction volumes are 0.5 mL and column void volume is about 6.5 mL. Two peaks of Aβ elute, one at the void volume and the second near the peak of the Aβd/t elution (about 12 ml). Upper plot shows the column calibration with points for chicken egg albumin (44 kDa), horse myoglobin (17 kDa) and vitamin B12 (1.35 kDa). Combined fractions of the high-n and low-n Aβ species used for the rod-induction assay in Figure 3B are underlined. Click here for file

Additional file 4 **The Aβd/t fraction remains stable for 48 h when incubated with neurons**. Immunoprecipitates from 7PA2 medium (IP positive controls on left) and from neuronal culture medium 48 h after treatment with Aβd/t, the equivalent fraction from NC medium, or the monomer fraction. The load volume on the right is equivalent to 0.4 mL of starting 7PA2 medium and the dimer/trimer bands are slightly less than what is contained in the 0.5 mL of starting medium showing that the d/t fraction is stable over the 48 h of culture. Click here for file

Additional file 5 **Measurement of adenoviral infection efficiency of cells in organotypic slices**. (A) Low magnification (4x objective) images of a hippocampal organotypic slice stained with DAPI, immunostained for cofilin, and imaged for GFP-expression after infection with adenovirus expressing GFP behind a CMV promoter. (B) Infection efficiency in organotypic slices, measured by the numbers of cells showing GFP fluorescence around a DAPI stained nucleus. Between 70-75% of the cells so examined were positive for GFP. (C) This panel was assembled to illustrate how we determined the percentage of infected cells. One region from a confocal plane of an image stack (60x objective) is shown. The areas surrounding nuclei within this plane were examined for GFP expression, which had to be above a threshold level to be counted as positive. Nuclei were often above or below the optical section containing the GFP and we used a Z stack to obtain the most accurate counts. Hundreds of nuclei across different areas were examined to obtain the infection efficiency. Click here for file
