# Supplementary figures and images for: Amyloid beta dimers/trimers potently induce cofilin-actin rods that are inhibited by maintaining cofilin-phosphorylation
Source: Mol Neurodegener. 2011 Jan 24;6:10. doi: 10.1186/1750-1326-6-10 (PMC3037337; doi:10.1186/1750-1326-6-10)

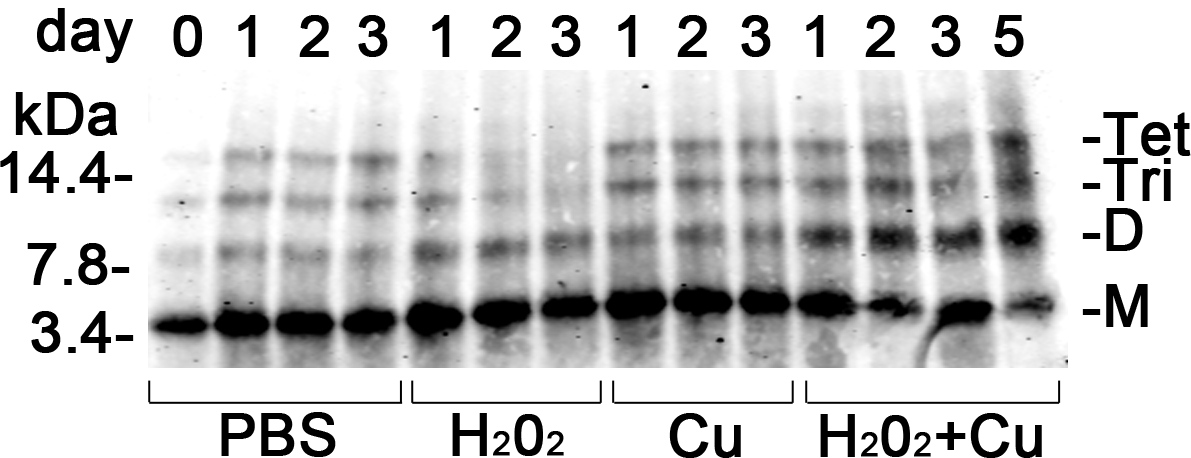

Supplement: Additional file 2 — Western blot showing time course of oxidative changes in synthetic human Aβ1-42 incubated under different conditions to generate SDS-stable dimers and higher oligomers. Synthetic human Aβ was dissolved to 5 μM directly into PBS alone or PBS containing 250 μM hydrogen peroxide, 25 μM CuCl2, or peroxide plus CuCl2, incubated at 37°C, and aliquots were removed at 1 day intervals for the times shown to examine the species present by Western blotting. After transfer, the blotting membrane was heated to boiling to expose the epitopes for detection with the 6E10 antibody. [file 1750-1326-6-10-S2.DOC]

**
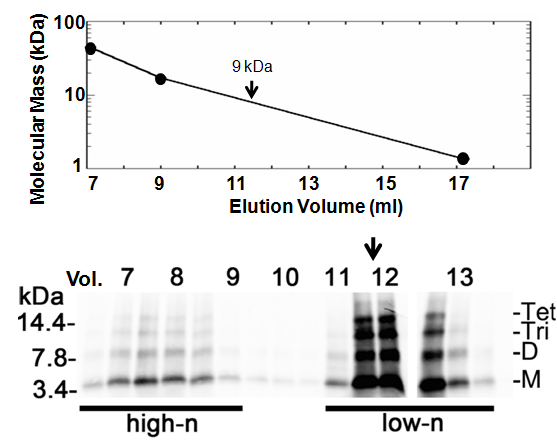
**

Supplement: Additional file 3 — Western blot showing fractions of gel filtration column of Cu2+-peroxide-treated synthetic human Aβ1-42. Aβ Western blot (6E10 antibody) of gel filtration fractions from a single Superdex75 (10/30 HR) column run at a flow rate of 0.5 mL/min and loaded with 1 mL of the Cu2+-peroxide-treated synthetic human Aβ1-42 after 5 days of incubation. Fraction volumes are 0.5 mL and column void volume is about 6.5 mL. Two peaks of Aβ elute, one at the void volume and the second near the peak of the Aβd/t elution (about 12 ml). Upper plot shows the column calibration with points for chicken egg albumin (44 kDa), horse myoglobin (17 kDa) and vitamin B12 (1.35 kDa). Combined fractions of the high-n and low-n Aβ species used for the rod-induction assay in Figure 3B are underlined. [file 1750-1326-6-10-S3.DOC]
