# Supplementary material for: Amyloid beta dimers/trimers potently induce cofilin-actin rods that are inhibited by maintaining cofilin-phosphorylation
Source: Mol Neurodegener. 2011 Jan 24;6:10. doi: 10.1186/1750-1326-6-10 (PMC3037337; doi:10.1186/1750-1326-6-10)
Supplement: Additional file 4 — The Aβd/t fraction remains stable for 48 h when incubated with neurons. Immunoprecipitates from 7PA2 medium (IP positive controls on left) and from neuronal culture medium 48 h after treatment with Aβd/t, the equivalent fraction from NC medium, or the monomer fraction. The load volume on the right is equivalent to 0.4 mL of starting 7PA2 medium and the dimer/trimer bands are slightly less than what is contained in the 0.5 mL of starting medium showing that the d/t fraction is stable over the 48 h of culture. [file 1750-1326-6-10-S4.DOC]

mL equivalent 0.4 mL equivalent

1 0.5 0 d/t std d/t NC M

Trimer

Dimer

Monomer

IP positive control IP Media after 48 h

culture
