# Supplementary material for: Amyloid beta dimers/trimers potently induce cofilin-actin rods that are inhibited by maintaining cofilin-phosphorylation
Source: Mol Neurodegener. 2011 Jan 24;6:10. doi: 10.1186/1750-1326-6-10 (PMC3037337; doi:10.1186/1750-1326-6-10)
Supplement: Supplementary file 3 [file 1750-1326-6-10-S1.DOC]

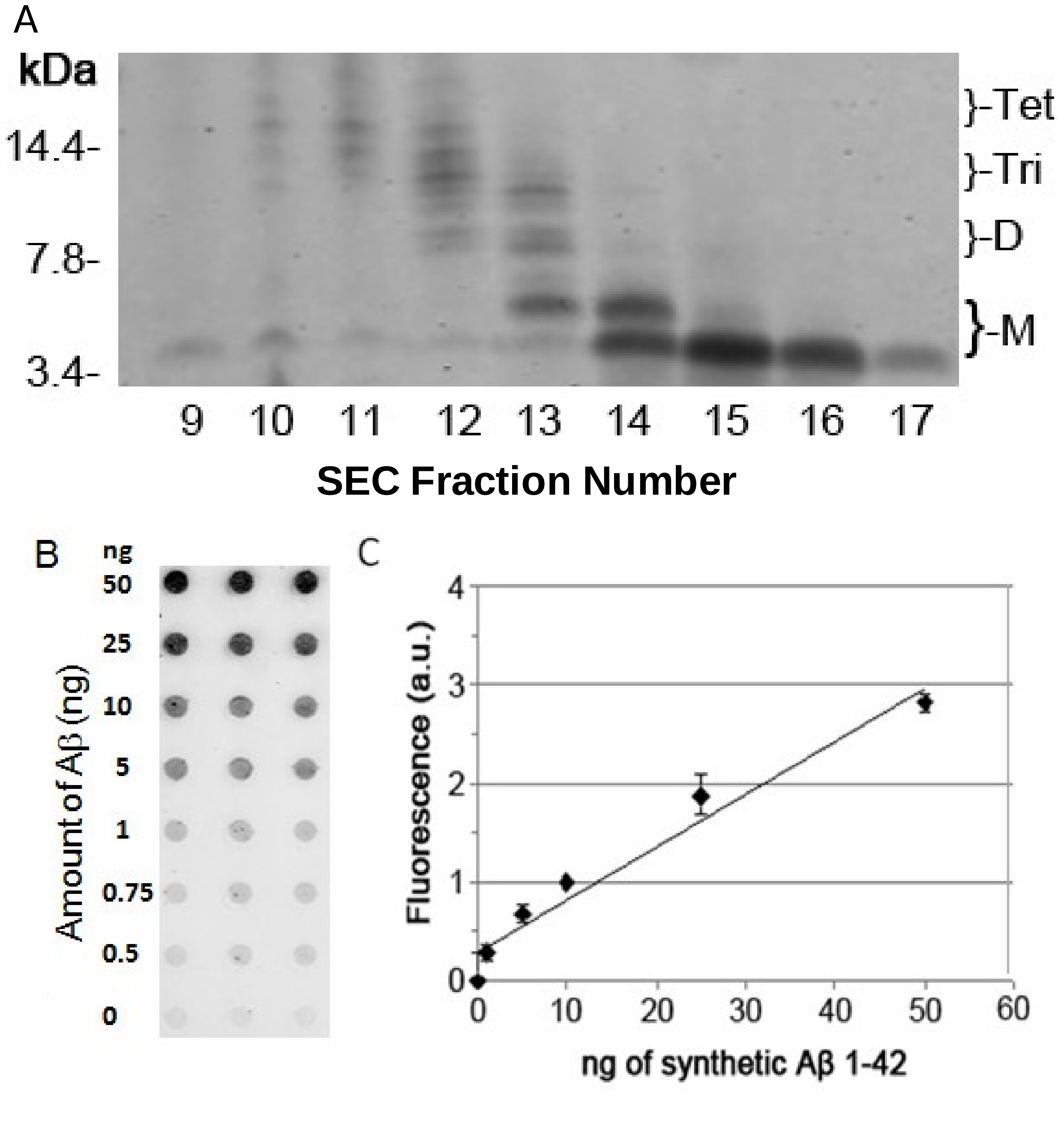
A
kDa
14.4-
7.8-
3.4-
}-Tet
}-Tri
}-D
}-M
9 10 11 12 13 14 15 16 17
SEC Fraction Number
B
ng
50
25
10
5
1
0.75
0.5
0
Amount of Aβ (ng)
C
Fluorescence (a.u.)
0 1 2 3 4
0 10 20 30 40 50 60
ng of synthetic Aβ 1-42
