# Supplementary material for: Amyloid beta dimers/trimers potently induce cofilin-actin rods that are inhibited by maintaining cofilin-phosphorylation
Source: Mol Neurodegener. 2011 Jan 24;6:10. doi: 10.1186/1750-1326-6-10 (PMC3037337; doi:10.1186/1750-1326-6-10)
Supplement: Supplementary file 4 [file 1750-1326-6-10-S5.DOC]

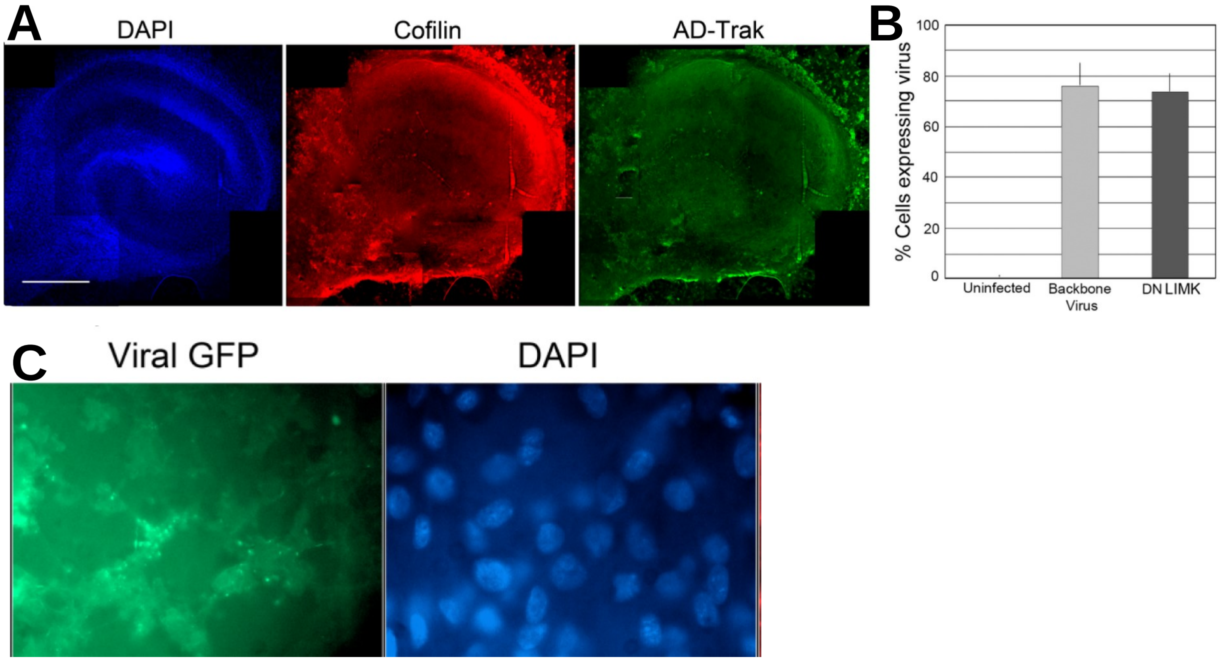
A
DAPI
Cofilin
AD-Trak
B
% Cells expressing virus
100
80
60
40
20
0
Uninfected
Backbone Virus
DN LIMK
C
Viral GFP
DAPI
